# Supplementary material for: The genome sequence of the common awl robberfly, Neoitamus cyanurus (Loew, 1849)
Source: Wellcome Open Res. 2024 Oct 17;9:289. Originally published 2024 May 22. [Version 2] doi: 10.12688/wellcomeopenres.22253.2 (PMC11494275; doi:10.12688/wellcomeopenres.22253.2)
Supplement: Peer review history

# Reviewer report: Reviewer response for version 2

**DOI:** 10.21956/wellcomeopenres.25629.r107566

**Published:** 2024-10-30

**Authors:** Jason Charamis

**Affiliations:** 1 Foundation for Research and Technology - Hellas, Irákleion, Greece

This manuscript describes the sequencing, assembly and annotation of the common awl robberfly, *Neoitamus cyanurus* genome. This is a great resource in the fields of arthropod ecology and predation and the inclusion of a genome annotation, dramatically increases its value and usability from researchers all over world.

 I have two minor points, which are not essential for publication:

 1. The mean value should be rounded and the metric (bp) should be added at the end of the sentence: 'The average transcript length is 12,082.69.'

 2. No quality assessment has been performed for the genome annotation. Running a BUSCO analysis on the predicted gene sets would provide valuable insights into the completeness and quality of the annotation.

Are sufficient details of methods and materials provided to allow replication by others?

Yes

Is the rationale for creating the dataset(s) clearly described?

Yes

Are the datasets clearly presented in a useable and accessible format?

Yes

Are the protocols appropriate and is the work technically sound?

Yes

Reviewer Expertise:

Arthropod Genomics, Bioinformatics

I confirm that I have read this submission and believe that I have an appropriate level of expertise to confirm that it is of an acceptable scientific standard.

# Reviewer report: Reviewer response for version 2

**DOI:** 10.21956/wellcomeopenres.25629.r105812

**Published:** 2024-10-21

**Authors:** Manee M Manee

**Affiliations:** 1 King Abdulaziz City for Science and Technology, Riyadh, Saudi Arabia

I am happy with the current form of the latest version of the manuscript.

Are sufficient details of methods and materials provided to allow replication by others?

Yes

Is the rationale for creating the dataset(s) clearly described?

Yes

Are the datasets clearly presented in a useable and accessible format?

Yes

Are the protocols appropriate and is the work technically sound?

Yes

Reviewer Expertise:

bioinformatics, genomics, and evolutionary biology

I confirm that I have read this submission and believe that I have an appropriate level of expertise to confirm that it is of an acceptable scientific standard.

# Reviewer report: Reviewer response for version 2

**DOI:** 10.21956/wellcomeopenres.25629.r105811

**Published:** 2024-10-18

**Authors:** Frances Swift

**Affiliations:** 1 The University of Edinburgh, Edinburgh, Scotland, UK

The only comment I have would be that again the regions on the Hi-C map (Figure 5) that don't appear to have long distance connectivity with the other regions if the authors could comment explaining some potential reasons for this/talk about the quality of the HiC data I think it could improve the genome note but not required for it to be publishable. I do appreciate that the authors have included clearer methodology on how the scaffolding was done with the HiC data in the first place. No further comments than that.

Are sufficient details of methods and materials provided to allow replication by others?

Yes

Is the rationale for creating the dataset(s) clearly described?

Yes

Are the datasets clearly presented in a useable and accessible format?

Yes

Are the protocols appropriate and is the work technically sound?

Partly

Reviewer Expertise:

Structural variation analysis in insect genomes

I confirm that I have read this submission and believe that I have an appropriate level of expertise to confirm that it is of an acceptable scientific standard.

# Reviewer report: Reviewer response for version 1

**DOI:** 10.21956/wellcomeopenres.24530.r101302

**Published:** 2024-10-10

**Authors:** Frances Swift

**Affiliations:** 1 The University of Edinburgh, Edinburgh, Scotland, UK

The authors describe the genome of an individual female *Neoitamus cyanurus *caught in Wytham woods, Oxfordshire. They report a genome with a total sequence length of 365.5 megabases. 95 contings were scaffolded into 16 scaffolds using HiC of which 10 represent chromosomes. No X chromosome was assembled due to insufficient evidence. Whilst not fully phased a single haplotype assembly was created, and scaffolds associated with a secondary haplotype were also deposited. Figure 3 shows little to no contamination in the genome assembly. The scaffold N50 length was reported as 42.7 mb. A mitochondrial genome of length 16.63 kb was also assembled. The BUSCO score of 96.2% and the other assembly statistics presented in Table 1 assure that the deposited genome is high quality.

 Whilst there are no quality concerns about the genome, this report could benefit from an author statement regarding how reliably the HiC data was used in the mapping as from Figure 5, there appear to be regions scaffolded into chromosomes that don’t show long-distance connectivity. Hence my partly answer for the question ‘Are the protocols appropriate and is the work technically sound’.

Are sufficient details of methods and materials provided to allow replication by others?

Yes

Is the rationale for creating the dataset(s) clearly described?

Yes

Are the datasets clearly presented in a useable and accessible format?

Yes

Are the protocols appropriate and is the work technically sound?

Partly

Reviewer Expertise:

Structural variation analysis in insect genomes

I confirm that I have read this submission and believe that I have an appropriate level of expertise to confirm that it is of an acceptable scientific standard.

# Reviewer report: Reviewer response for version 1

**DOI:** 10.21956/wellcomeopenres.24530.r98382

**Published:** 2024-09-25

**Authors:** Manee M Manee

**Affiliations:** 1 King Abdulaziz City for Science and Technology, Riyadh, Saudi Arabia

This study presents the first genome assembly of *Neoitamus cyanurus*, the common awl robber fly. The genome span is 365.5 megabases and is scaffolded into ten chromosomal pseudomolecules. The assembly achieved a BUSCO completeness score of 96.2%, with 95.3% single-copy and 0.9% duplicated orthologs, reflecting the high genome quality. The genome assembly of *Neoitamus cyanurus* provides valuable genetic insights into robberfly evolution and predatory traits, contributing to broader efforts to understand biodiversity through the Darwin Tree of Life Project. I strongly encourage the authors to proceed with annotating their genome assembly, as this will greatly enhance its utility for the broader scientific community.

Are sufficient details of methods and materials provided to allow replication by others?

Yes

Is the rationale for creating the dataset(s) clearly described?

Yes

Are the datasets clearly presented in a useable and accessible format?

Yes

Are the protocols appropriate and is the work technically sound?

Yes

Reviewer Expertise:

bioinformatics, genomics, and evolutionary biology

I confirm that I have read this submission and believe that I have an appropriate level of expertise to confirm that it is of an acceptable scientific standard, however I have significant reservations, as outlined above.
